# Dynamic Transcriptional Landscape of *Mycobacterium smegmatis* under Cold Stress

**DOI:** 10.3390/ijms241612706

**Published:** 2023-08-11

**Authors:** Artem S. Grigorov, Yulia V. Skvortsova, Oksana S. Bychenko, Leonid V. Aseev, Ludmila S. Koledinskaya, Irina V. Boni, Tatyana L. Azhikina

**Affiliations:** Shemyakin and Ovchinnikov Institute of Bioorganic Chemistry, Russian Academy of Sciences, Moscow 117997, Russia

**Keywords:** stress resistance, survival, RNA-seq, cold shock response, adaptation, mycobacteria

## Abstract

Bacterial adaptation to cold stress requires wide transcriptional reprogramming. However, the knowledge of molecular mechanisms underlying the cold stress response of mycobacteria is limited. We conducted comparative transcriptomic analysis of *Mycobacterium smegmatis* subjected to cold shock. The growth of *M. smegmatis* cultivated at 37 °C was arrested just after exposure to cold (acclimation phase) but later (by 24 h) was resumed at a much slower rate (adaptation phase). Transcriptomic analyses revealed distinct gene expression patterns corresponding to the two phases. During the acclimation phase, differential expression was observed for genes associated with cell wall remodeling, starvation response, and osmotic pressure stress, in parallel with global changes in the expression of transcription factors and the downregulation of ribosomal genes, suggesting an energy-saving strategy to support survival. At the adaptation phase, the expression profiles were recovered, indicating restoration of the processes repressed earlier. Comparison of transcriptional responses in *M. smegmatis* with those in other bacteria revealed unique adaptation strategies developed by mycobacteria. Our findings shed light on the molecular mechanisms underlying *M. smegmatis* survival under cold stress. Further research should clarify whether the discovered transcriptional mechanisms exist in other mycobacterial species, including pathogenic *Mycobacterium tuberculosis*, which could be important for transmission control.

## 1. Introduction

The immense diversity of microbes, which can inhabit almost every environmental niche on Earth, is due to their high adaptability to fluctuating environmental conditions, including extreme temperatures. The detrimental effects of cold stress include decreased membrane fluidity, slowdown of metabolism, increased stability of secondary RNA structures, and topological alterations in DNA [1,2]. 

Mycobacteria, with its most important representative *Mycobacterium tuberculosis* (MTB), a causative agent of tuberculosis, can survive in diverse habitats [3,4,5]. Thus, during the transmission period between individuals, *M. tuberculosis* can resist sharp changes in ambient temperature, which accounts for its evolutional success and suggests a sophisticated network of adaptive cellular and molecular mechanisms. However, although mycobacteria are one of the most extensively studied groups of microorganisms, little is known about their physiological and molecular reactions to cold stress. To our knowledge, there is only one study on the effects of low temperatures on *Mycobacterium smegmatis* which has revealed that a sharp temperature decrease is associated with a protracted lag phase (21–24 h) occurring before the resumption of cell growth (but at a much slower rate) and the induction of some measurable cellular activities [6]. Rustad and colleagues [7] demonstrated that modest (20ºC) cold shock substantially greatly stabilizes *M. tuberculosis* mRNA.

*M. smegmatis* is a fast-growing non-pathogenic bacterium that can survive in various habitats, including soil and aquatic conditions [8]. *M. smegmatis* encodes numerous conserved mycobacterial protein orthologs and has cell architecture and physiology similar to those of other Mycobacterium species, including MTB; therefore, *M. smegmatis* is frequently used as a model organism to study adaptive mechanisms developed by pathogenic mycobacteria [9]. In particular, understanding the *M. smegmatis* response to cold stress can shed light on the strategies employed by MTB to survive and spread in low-temperature environments and help to identify prospective targets for developing effective measures to interrupt MTB transmission.

Over the last decade, functional genomics has emerged as a powerful tool to study gene expression profiles in bacteria under various stresses and disclose microbial adaptation strategies to adverse conditions [10,11]. To gain insights into the regulatory mechanisms triggered by cold shock in mycobacteria, in this study, we performed, for the first time, comprehensive transcriptional analysis of *M. smegmatis* exposed to cold stress and determined key functional gene groups associated with response to low temperatures. Our findings should bridge the gap in our understanding of the molecular mechanisms underlying the cold stress adaptation of *M. smegmatis* and possibly other mycobacterial species. 

## 2. Results

### 2.1. *M. smegmatis* Growth under Cold Stress

**Figure 1:**
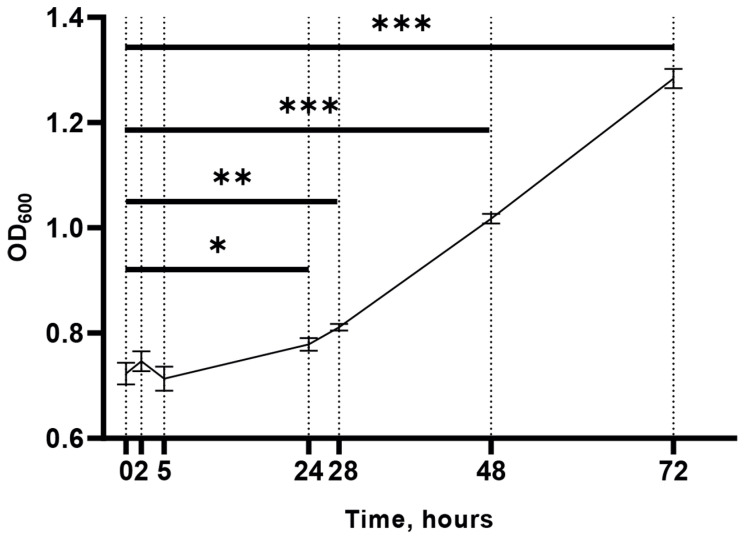
Growth curve of *M. smegmatis* under cold stress conditions (15°C). The data are presented as the mean ± SD of three independent experiments; * *p* < 0.05, ** *p* < 0.01, and *** *p* < 0.001 indicate statistically significant differences with control (H0). OD_600_, optical density at 600 nm.

To determine appropriate time points for subsequent transcriptomic analysis, we analyzed the growth kinetics of *M. smegmatis* exposed to cold stress (15 °C). The results revealed that during the onset of cold stress response, the first statistically significant increase in the optical density of the cell culture was observed only at 24 h, indicating the cessation of cell division during the acclimation period (Figure 1), which is consistent with previous findings [6]. 

For RNA-seq analysis, we chose the following time points: 0 h (H0, prior to cold exposure); 2 and 5 h (H2 and H5, respectively, representing transient responses to cold, or the acclimation stage); and 24 h (H24, corresponding to the resumption of cell growth or the adaptation stage).

### 2.2. Transcriptional Landscape

**Figure 2:**
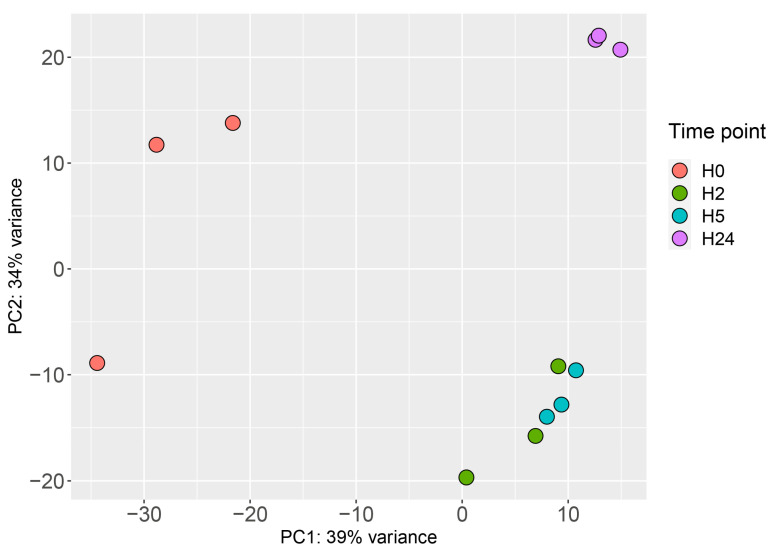
Time-dependent clustering of RNA-seq data revealed by PCA.

To perform time-dependent transcriptional profiling in *M. smegmatis*, bacterial cultures were subjected to RNA-seq at each time point. The mapping statistics is shown in Suppl. Table S1. Principal component analysis (PCA) of the obtained data revealed the presence of three distinct clusters: control (before stress, H0); the acclimation stage (H2 and H5); and the adaptation stage (H24) (Figure 2).

**Figure 3:**
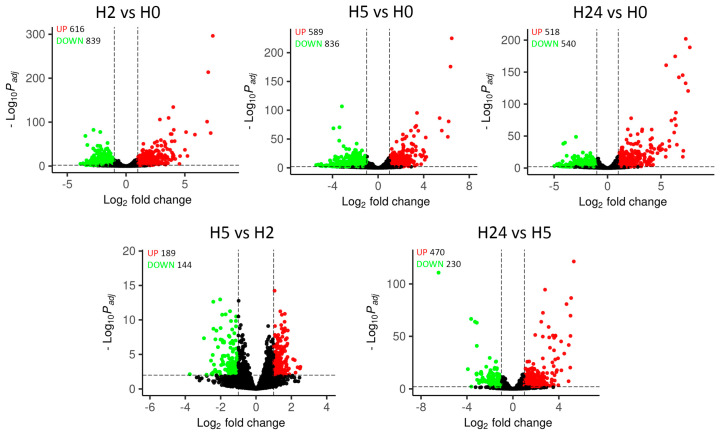
Volcano plots of *M. smegmatis* DEGs. Upper panels show expression changes at each time point compared to control (H0) and lower panels—those at each time point compared to the previous one. DEGs (|log2FC| ≥ 1, p.adj < 0.01) are shown in red (upregulated) and green (downregulated).

Using the DESeq2 software package, we identified genes differentially expressed at each time point compared to control (H2, H5, or H24 vs. H0) and in dynamics (H2 vs. H0, H5 vs. H2, and H24 vs. H5). The results indicated that cold exposure caused changes in RNA level in a large number of genes; thus, 1,455 genes (nearly a quarter of the *M. smegmatis* genome) showed differential expression (FC > 2) as early as 2 h after cold stress (Figure 3). The overall expression pattern was consistent with the slowdown of cell metabolism (Figure 1) as the number of the downregulated DEGs (839) was greater than that of the upregulated ones (616). The lists of differentially expressed genes for all comparisons are presented in Suppl. Table S2.

**Figure 4:**
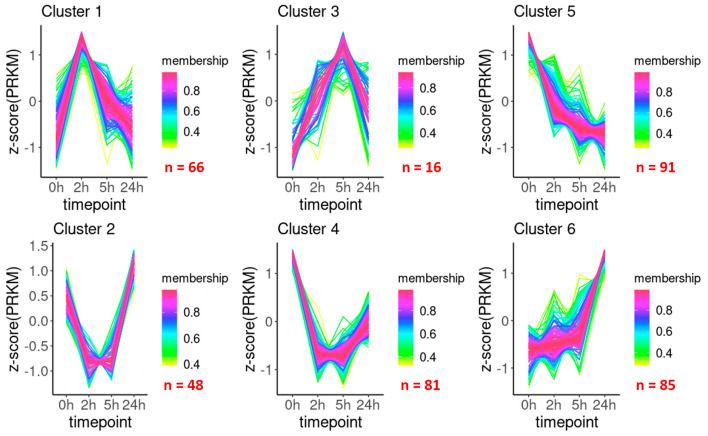
Cluster analysis of time-dependent gene expression patterns in *M. smegmatis* after cold exposure. Clustering was performed using TCseq software package. The y-axis indicates the Z-score calculated using reads per kilobase per million (RPKM); membership values indicate the degree of cluster separation. The number of genes included in each cluster according to the selected threshold (0.75) is indicated at the bottom right of each graph.

Then, we analyzed the acquired transcriptomes using the TCseq software package and grouped DEGs based on their change patterns. Membership values denoting the association of each gene to a specific cluster are presented in Suppl. Table S3 (sheet 1, “TCseq analysis”). We considered a gene to belong to a particular cluster if the membership value was greater than 0.75. According to cluster shapes, which suggest functional roles of the contained genes, six clusters were identified (Figure 4). Genes from clusters 1 and 3 were transiently upregulated at the acclimation stage (2 and 5 h) and then downregulated at the adaptation stage (24 h), indicating the involvement of these genes in the early response of *M. smegmatis* to low temperatures. On the other hand, the expression of genes in clusters 2 and 4 was sharply downregulated during the acclimation stage but was recovered (albeit at varying degrees) by 24 h, when cells resumed active division. Genes generally downregulated by cold temperature were grouped in cluster 5, whereas those consistently upregulated were grouped in cluster 6 (Figure 4), indicating their involvement in the immediate and long-term response, respectively, of *M. smegmatis* to cold stress. 

Overall, these results revealed that the transcriptional changes occurring in *M. smegmatis* after cold exposure could be divided into two stages roughly corresponding to the growth kinetics. The first one represented rapid reaction (H2 and H5) revealed as a general reduction of transcriptional activity (clusters 1 and 3) despite the induction of certain genes, whereas the second one represented long-term adaptation (H24) characterized by the re-activation of genes downregulated at the first stage (clusters 2 and 4) and induction of specific genes required to support cell growth under prolonged stress (cluster 6). It should also be noted that the observed expression changes could occur very rapidly; among cluster 1 genes, a significant number exhibited a sharp increase in expression at 2 h and an equally significant decrease at 5 h (Figure 4). Such an immediate transcriptional response to environmental conditions seems to be characteristic of mycobacteria [12].

**Figure 5:**
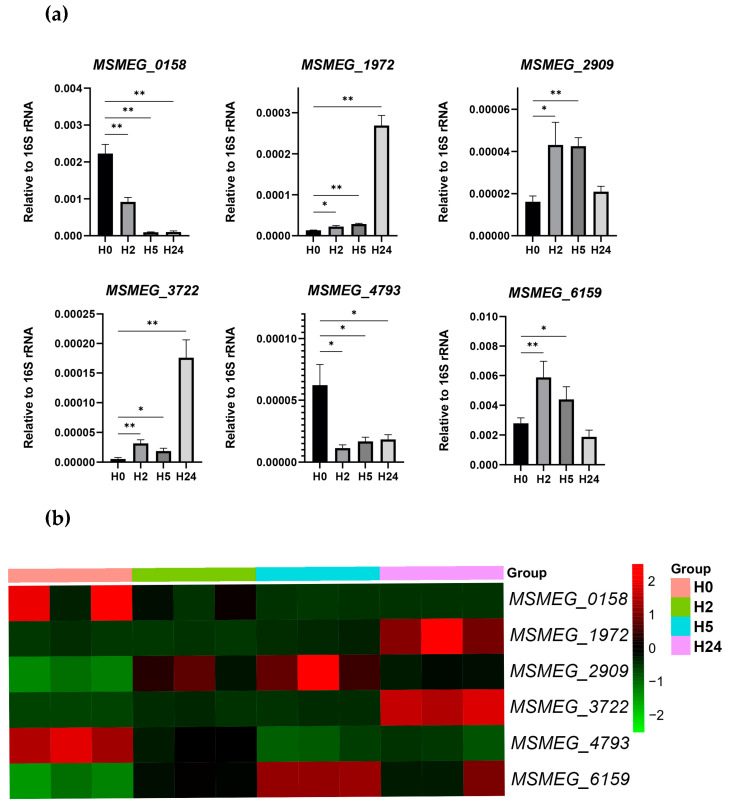
Analysis of selected DEGs. (**a**) Validation of DEGs via qRT-PCR. mRNA level was determined at each time point (H0, H2, H5, and H24) and normalized to that of 16S rRNA. The data are presented as the mean and SD of three biological replicates for each time point; * *p* < 0.05 and ** *p* < 0.01 compared to H0. (**b**) Heatmap of normalized RNA-seq data for the selected DEGs was constructed using https://www.bioinformatics.com.cn/en, accessed on 17 May 2023.

The transcriptomic changes detected by RNA-seq were validated by qRT-PCR. The results confirmed that the transcription profiles of the selected genes (MSMEG_0158, MSMEG_1972, MSMEG_2909, MSMEG_3722, MSMEG_4793, and MSMEG_6159) obtained by qRT-PCR (Figure 5a) coincided with those obtained by RNA-seq (Figure 5b).

To reveal the functional enrichment of genes in the clusters, we performed Gene Onthology analysis. These results are given in the Suppl. Table S3 (sheet 2, “GO analysis”). However, due to the current lack of comprehensive annotations for many genes in *M. smegmatis*, we also selected functional groups of genes, based on their existing description. The most important groups of DEGs potentially involved in the cold stress response are listed in Table 1 and Table 2.

### 2.3. Small Non-Coding RNAs 

Although several studies have attempted to delineate the non-coding transcriptome of *M. smegmatis* [13,14], there is currently no accepted annotation for this mycobacterial species. Here, we attempted to annotate the *M. smegmatis* small non-coding RNA (sRNA) genes. The boundaries of potential sRNA genes were annotated for each sample and then combined into a single annotation, according to which the counts for each gene were calculated using total transcriptome data, and changes in gene expression relative to H0 were analyzed. We named the detected sRNAs in accordance with the nomenclature based on proximity to the upstream gene proposed by Lamichhane et al [15].

In total, we identified 43 cis- and 13 trans-encoded sRNAs (Suppl. Table S4). Among them, four sRNAs (ncMSMEG10373B, ncMSMEG11192, ncMSMEG15794c, ncMSMEG1286) overlapped with the coordinates of previously annotated sRNAs IGR-2, IGR-3, IGR-5, and AS-5, respectively [13]. Although according to our data, these sRNA genes appeared to be longer than previously reported, it could be due to the presence of alternative transcripts detected by northern blotting [13]. Our transcriptomes also included one of the most studied mycobacterial sRNAs, Ms1 (also known as 6S), which was first identified in *M. smegmatis* by Pánek and coauthors [16]; the results were consistent with previous findings in regard to the annotated Ms1 length (approximately 300 nt) but differed from those in regard to the transcriptional start point by three nucleotides [17]. Transcription coverage profiles of several antisense and intergenic sRNAs examples are given in the Suppl. Figure S1. The predicted secondary structures of intergenic sRNAs are presented in the Suppl. Figure S2.

## 3. Discussion

In this study, we demonstrated that cold stress altered the growth kinetics of *M. smegmatis*, which showed a protracted lag phase (about 24 h) before the resumption of logarithmic growth after 24 h, thus confirming previous findings [6]. Comparative analysis of RNA-seq data at the four time points revealed that the *M. smegmatis* adaptation to cold stress was a two-stage process. Thus, at the first stage (2–5 h post-shock), a set of genes were upregulated despite the general downregulation of transcriptional activity, which resulted in cessation of cell division; the opposite pattern was observed at the second stage (24 h post-shock), when the genes activated earlier were repressed and those repressed earlier were re-activated, which led to the re-start of cell growth. These results indicate that in *M. smegmatis*, different sets of genes are induced during the cold acclimation and adaptation stages. 

Below, we discuss the most important functional groups of genes that could be associated with *M. smegmatis* response to low temperatures.

### 3.1. RNA Chaperones

Cold shock proteins (CSPs) bind to and stabilize RNA and DNA architecture, preventing the formation of secondary structures which could interfere with transcription and translation. CSPs have been first identified as proteins significantly upregulated in *E. coli* after cold stress [18]. To date, nine proteins belonging to the cold shock protein family in *E. coli* are known (CSPs from CspA to CspI) [19]. Out of them, only five (CspA, B, E, G, I) are cold-shock-induced, but remaining four (C, D, F, H) are not induced by cold [20].

*M. smegmatis* has two proteins containing cold shock domains: CspA (MSMEG_6159) and CspB (MSMEG_5696); the former shares 91% and 92.5% sequence homology with CspA of *E. coli* and MTB, respectively. However, although the *M. smegmatis* CspA is functionally active and can bind oligonucleotides [21], we detected only slight transcriptional activation of cspA (about 2-fold) and none of cspB (Figure 5), in contrast to *E. coli*, where cspA is upregulated 25-fold after cold exposure [22]. 

Our findings suggest that the functional activity of Csps in *M. smegmatis* is not directly related to cold adaptation, which is unusual among microorganisms, in which Csp induction is one of the most conserved adaptive mechanisms. It should be noted that mycobacteria have a high GC content (67.4% in *M. smegmatis*), which could lead to the formation of secondary RNA structures [23] prevented by RNA chaperones such as Csps that support proper RNA folding and efficient transcription, translation, and RNA processing. Therefore, it can be suggested that in mycobacteria, Csps are involved in the physiological maintenance under normal conditions rather than in cold stress response as is the case in *E. coli*. However, this hypothesis needs experimental support. 

Among the other genes encoding RNA chaperones in *M. smegmatis*, MSMEG_1930 and MSMEG_1540 (DEAD/DEAH-box RNA helicases) were sharply upregulated early after cold exposure and downregulated thereafter, which is consistent with their role in the cold response of bacteria [24,25].

### 3.2. DNA Conformation and Repair Genes

The topology of bacterial chromosomes is known to be altered by low temperatures. Thus, in *E. coli*, cold stress changes the supercoiling state of DNA and, consequently, gene expression patterns [26]. Negative supercoiling of *E. coli* DNA is hypothesized to promote the transcription of cold shock genes and adaptation to low temperatures [27]. Gyrases (gyrA, gyrB), topoisomerase (topA), and histone-like protein (hupB) induced by low temperatures are involved in the regulation of DNA topology and repair in *E. coli*, *B. subtilis*, and lactic acid bacteria [20,24,28,29]. 

Our results also revealed the upregulation of *M. smegmatis* genes encoding type-1 topoisomerase (MSMEG_1784) and histone-like protein hup (MSMEG_2389) during the first hours of cold exposure (Table 1), which is in agreement with a previous study showing the induction of MSMEG_2389 transcription and translation during the acclimation period [6], suggesting a role in cold stress adaptation. The other early induced genes were those of the ruvABC complex, which in *E. coli* facilitates branch migration and resolves Holliday structures during homologous recombination [30]. At the later (adaptation) stage, the MSMEG_6083 gene, which encodes a protein involved in base excision repair and prevention of mutagenesis in the mycobacterial genome [31], was upregulated.

### 3.3. Osmoprotectant (Compatible Solute) Genes

**Figure 6:**
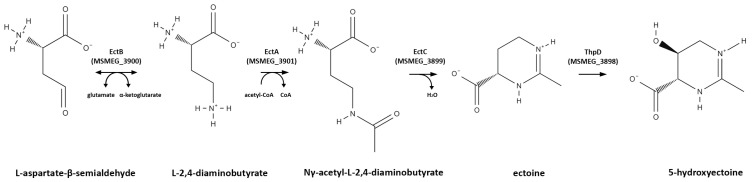
The biosynthetic pathway of ectoine and hydroxyectoine, adapted from [32]. In the early response to cold stress, genes encoding all proteins involved in this pathway have been found.

In *M. smegmatis*, we observed immediate activation of all four genes (MSMEG_3898, MSMEG_3899, MSMEG_3900, and MSMEG_3901) associated with the synthesis of the osmoprotectant ectoine and its hydroxylated derivative, hydroxyectoine [32], which continued at the late stage (Table 1), suggesting that ectoine plays a key role in the adaptation of mycobacteria to low temperatures. The biosynthetic pathway of ectoine and hydroxyectoine is depicted in Figure 6. In many bacterial species, ectoine acts as a highly effective osmoprotectant against drying, freezing, and severe osmotic stress, and has been shown to also be involved in the cold stress response of *E. coli* [33], Vibrio anguillarum [34], and Virgibacillus pantothenticus [35].

The glycine betaine transporter (opuD) gene (MSMEG_1883), associated with the adaptation of *B. subtilis* to low temperatures [36], was also upregulated in *M. smegmatis* at the early stage of cold exposure (2 and 5 h).

Genes associated with choline metabolism (MSMEG_5967, MSMEG_5305, and MSMEG_5944) were also activated; among them, the first two were upregulated both at the early and late stage of cold response (Table 1). Choline as a precursor in the synthesis of glycine betaine confers osmotic tolerance to bacteria [37,38]. However, the functional activity of these genes is still poorly understood, and their direct role in the cold stress adaptation of mycobacteria requires further investigation.

### 3.4. Transcriptional Regulator and Sigma Factor Genes

Consistent with large-scale rearrangements in the *M. smegmatis* transcriptome, we observed the induction of more than 50 transcriptional regulators (annotation was retrieved from the MycoBrowser database, https://mycobrowser.epfl.ch/, accessed on 5 December 2022) as early as 2 h after cold exposure, and the inhibition of 25 of them at the later time points. The genes with the most pronounced changes are listed in Table 2.

**Table 2:** DEGs encoding transcription factors.

Gene	Expression Pattern (Fold Change vs. H0)			Regulatory Function
	H2	H5	H24	
*MSMEG_6903*	16.06	18.84	7.29	Inositol metabolism [39]
*MSMEG_0120*	4.33	2.64	N/A	Lipid metabolism and cellular redox balance [40]
*MSMEG_5174*	3.89	3.80	4.95	Purine metabolism [41]; the MTB homologue (*Rv1152*) is strongly expressed in persisting and starved cells [42,43]
*MSMEG_1747* (*sigH7*)	3.81	N/A	2.03	Response to starvation, heat shock and oxidative stress [44]; Interestingly, in a previous study, the response of this sigma factor to cold stress was not detected [44]
*MSMEG_2694*	3.78	2.17	N/A	Redox stress; control of σH and σE regulons and Clp protease expression [45]
*MSMEG_2794*	2.60	2.20	N/A	Biosynthesis of mycolic acids through repression of the kas-operon [46]; the MTB homolog (*Rv0494*) is induced by starvation and hypoxia [47,48]
*MSMEG_6077* (*carD*)	2.04	N/A	N/A	Global transcriptional regulation in *M. smegmatis* and MTB; direct interaction with RNA polymerase, transcriptional control of over 350 gene; critical for stress response [49]
*MSMEG_1995*	2.24	N/A	N/A	MTB homolog (*Rv0165c*) represses the mammalian cell entry 1 (*mce1*) operon [50]
*MSMEG_0156* (*oxyS*)	N/A	0.17	0.27	Response to oxidative stress [51]
*MSMEG_0937* (*regX3*)	0.34	0.35	N/A	Response to phosphate starvation [52]
*MSMEG_5872* (*phoP*)	0.17	0.27	N/A	Response to oxidative stress and hypoxia [53]

Upregulated and downregulated genes are highlighted red and green, respectively. N/A, no change.

It should be noted that the expression of SigF, which is one of the most extensively studied mycobacterial sigma factors involved in responses to oxidative stress and heat shock, the synthesis of cell wall components, and metal ion homeostasis [54,55], was not changed in *M. smegmatis* after cold exposure. However, at least 50 genes of the SigF regulon [54,55] were repressed at the early stage by more than 2-fold; among them, 27 belonged to clusters 2 and 4 comprising genes which restored expression at the late stage. The anti-SigF antagonist RbsW (MSMEG_1787) was also downregulated by more than 8-fold at the early stage, which could lead to an increase in the concentration of free SigF antagonist and removal of the sigma factor from its active state; however, by 24 h, the transcription of rbsW returned to its initial level. Thus, unlike other adverse conditions, cold is probably not a kind of stress that requires the activity of the SigF regulon for adaptation.

### 3.5. Translation-Related Genes

In *E. coli*, low temperatures activate transient expression of the genes encoding ribosome-binding protein RbfA and a DEAD-box helicase CsdA involved in 16S RNA processing [56] and the biogenesis of the large ribosomal subunit [57], respectively, as well as those encoding translation initiation factors InfA, InfB, and InfC [58,59,60]. In *B. subtilis*, cold stress also induces the expression of initiation factors, RbfA and CsdA homologs, and other ribosomal genes [24]. 

In contrast, translation-related genes were not upregulated in *M. smegmatis* after cold exposure. The only activated gene was MSMEG_1878 (mycobacterial protein Y), involved in the hibernation of 70S ribosomes [61] and induced exclusively in the stationary growth phase [62]. MSMEG_1878 expression was upregulated as early as 2 h after cold stress (3.1-fold), reaching maximum by 24 h (5.97-fold). At the same time, a large number of ribosomal protein genes (>25) were downregulated at the early time points and regained the initial activity by 24 h.

Thus, it can be hypothesized that cold stress reduced the translation rates in *M. smegmatis* through ribosome hibernation and the transcriptional repression of ribosomal genes, which could be reactivated upon stress relief [63].

### 3.6. Genes Involved in Protein Turnover

We observed the upregulation of several heat-response genes (dnaK, grpE, dnaJ, groL, clpC3, htpX, and lon) involved in the refolding or removal of misfolded, damaged, and aggregated proteins (Table 1). Among them, dnaK, grpE, dnaJ, and groL, known to be associated with the adaptation of several bacteria to low temperatures [64,65,66], were induced rapidly and remained activated throughout the experiment, whereas protease genes (clpC3, htpX, and lon) were mostly induced at 2 h. Clp proteases have been implicated in the cold stress response of bacteria; thus, ClpB plays a role in the adaptation of cyanobacterium Synechococcus sp. strain PCC 7942 to permissive low temperatures [67]. However, the exact function of heat shock proteins in the cold stress adaptation of mycobacteria remains unclear.

### 3.7. Genes Related to Cell Wall and Lipid Composition

The structural and functional integrity of bacterial cell walls and membranes can be affected by low temperatures, resulting in decreased fluidity, increased rigidity, and altered permeability, which, in turn, can impair the function of membrane-associated enzymes, transporters, and receptors. Bacteria are known to modulate the structure of cell walls and membranes in response to cold stress by changing their lipid composition [68,69].

Consistent with this notion, in cold-exposed *M. smegmatis*, we observed an increase in the expression of several cyclopropane synthases (MSMEG_1350, MSMEG_0902, MSMEG_1205, MSMEG_3538, and MSMEG_1351) involved in the modification of mycolic acids (Table 1); some of these genes were mostly upregulated at 2 h and others at 24 h, suggesting control of the cell-wall lipid composition throughout the entire period of cold exposure. At least one of these genes (MSMEG_1351) has already been implicated in cold stress response of mycobacteria, where MSMEG_1351 catalyzes the formation of a new cyclopropanated lipid [70]. An increase in the synthesis of cyclopropane fatty acids (CFAs) in response to low temperatures has been also observed in other bacteria [71,72]. Although the exact role of CFAs is unknown, they are thought to be involved in the regulation of membrane fluidity. 

A much more common mechanism for modulating membrane fluidity is the desaturation of fatty acids [73], and our results revealed the upregulation of genes encoding *M. smegmatis* desaturases (MSMEG_1743, MSMEG_5773, and MSMEG_5248) in the first hours of stress exposure.

Among the slow-response genes (cluster 6) affecting the structure/composition of the cell membrane/wall, we detected MSMEG_6281 encoding cell wall hydrolase (MTB homolog Rv3717 [74]) and MSMEG_4727 encoding a protein involved in the synthesis of lipo-oligosaccharides [75].

### 3.8. Transporter Genes

We also observed significant changes in the expression of over 300 transporter genes annotated according to the TransportDB 2.0 database [76] (Suppl. Table S5). The most significant functional groups are the following:

- Transporters of osmoprotectants betaine and ectoine *MSMEG_1883* and *MSMEG_5368*, respectively, which were upregulated. This process likely reflects another pathway for the accumulation of osmoprotectants in the cell, in addition to the synthesis described earlier;
- Transporters of nutrients (amino acids, sugars, and ions), most of which were downregulated which may reflect a general slowdown in metabolic activity under cold stress—this could be a survival strategy, as slowing down metabolism reduces the energy demand and helps the cell to conserve resources;
- Efflux pumps, some of which were upregulated (multidrug efflux pump gene *MSMEG_6225* and daunorubicin export gene *MSMEG_6509*), which might indicate an increased sensitivity to the toxic stress under cold conditions, and others downregulated, probably representing a strategy for energy conservation;
- Lipid transporters demonstrate various patterns of cold response and distributed among all six clusters, reflecting dynamic changes in *M. smegmatis* lipid composition during cold stress adaptation. Among them, the genes of mycobacteria-specific *mce* operons, which encode ABC-like lipid transporters participating in cell wall remodeling [77], were downregulated at the acclimation stage, except for *MSMEG_6540*, which was upregulated. As described earlier, changes in lipid composition can affect the fluidity and integrity of the cell membrane, which are crucial for maintaining cell function during temperature changes. Alterations in the spectrum of lipid transporters is an integral part of this adaptive process.

### 3.9. sRNAs

Small non-coding RNAs are central players in the adaptation of mycobacteria to environmental changes. However, only a few sRNAs have been reported to be involved in the response of bacteria to low temperatures [78,79,80].

Cis-encoded sRNAs are transcribed from a DNA strand complementary to the target gene, thus providing an overlapping region necessary for RNA interaction. Among the cis-encoded RNAs annotated in *M. smegmatis*, three (ncMSMEG0671, ncMSMEG1286, ncMSMEG3950c) were induced by cold stress, whereas their potential target mRNAs (MSMEG_0671, MSMEG_1286, and MSMEG_3950) were reduced (Suppl. Table S2).

Among trans-encoded sRNAs, which show only partial complementarity to the target, Ms1 was upregulated in *M. smegmatis* by approximately 3.7-fold after 24 h of cold exposure. Ms1 is the most abundant among sRNAs in stationary-phase cultures of *M. smegmatis* [81]; it interacts with the RNA polymerase core, competing with sigma factors, and it is involved in the transcription of the RNA polymerase β and β′ subunits [17], thus controlling transcriptional activity and maintaining the pool of RNA polymerase molecules for the recovery from the stationary phase. It is likely that such processes also occur under cold stress, suggesting a similarity between transcriptional regulation at the stationary growth phase and in cold response.

To the best of our knowledge, our study represents the first attempt to describe changes in a non-coding bacterial transcriptome in response to cold stress. The data are also important for the development of a more comprehensive annotation of sRNA genes in *M. smegmatis*.

## 4. Materials and Methods

### 4.1. Bacterial Strains and Growth Conditions

*M. smegmatis* MC2 155 obtained from the bacterial collection of the Bach Institute of Biochemistry (Research Center of Biotechnology of the Russian Academy of Sciences, Moscow, Russia) was revived from a frozen stock after incubation in Nutrient Broth (Himedia, Mumbai, India) supplemented with 0.05% (*v*/*v*) Tween 80 (Panreac, Barcelona, Spain) at 37 °C in an orbital shaker-incubator ES-20 (Biosan, Riga, Latvia) at 200 rpm for 30 h.

For cold stress experiments, *M. smegmatis* cells were grown up to the mid-logarithmic phase (OD_600_ = 0.7) at 37 °C and then transferred to 15 °C and cultured in a shaking water bath (Innova 3100, New Brunswick Scientific, Edison, NJ, USA) at 200 rpm, with periodical monitoring of OD_600_. Aliquots of 10 ml were withdrawn at 0, 2 h, 5 h and 24 h time points after cold shock and used for total RNA extraction.

### 4.2. Total RNA Extraction

At each time point, 10 ml aliquots removed were centrifuged at 4000 rpm for 15 min at 4 °C; pellets were washed twice with fresh cold medium and centrifuged again. Total RNA was isolated by phenol–chloroform extraction and cell disruption using Bead Beater (BioSpec Products, Bartlesville, OK, USA) as described previously [7] and treated with Turbo DNase (Life Technologies, Carlsbad, CA, USA) to remove traces of genomic DNA. RNA quantity and purity were determined spectrophotometrically, and its integrity was assessed by electrophoresis in 1% agarose gels.

### 4.3. Isolation of sRNA Fraction

Total RNA samples were denatured in equal volumes of 2× RNA Gel Loading Dye (Thermo Fisher Scientific, Waltham, MA, USA) by heating at 70 °C for 5 min, chilled on ice, and subjected to denaturing polyacrylamide gel electrophoresis (PAGE) in 15% gels containing 7 M urea. Gels were stained with ethidium bromide, and the sRNA fraction (≤500 nucleotides) was carefully excised, transferred to a microcentrifuge tube, eluted by passive diffusion in 0.3 M NaCl at 37 °C overnight, and purified by phenol–chloroform extraction and ethanol precipitation. The concentration and quality of the purified sRNA samples were assessed using a NanoDrop 2000C spectrophotometer (Thermo Fisher Scientific, Waltham, MA, USA).

### 4.4. Generation of RNA-seq Libraries and Data Analyses

Total RNA was depleted of rRNA using the NEBNext® rRNA Depletion Kit (Bacteria) (NEB, Ipswich, MA, USA) and used to generate sequencing libraries with the NEBNext Ultra II Directional RNA Library Prep Kit (NEB) according to the manufacturer’s protocol. Sequencing was performed in triplicate as pair-ended 150 nt reads using Illumina NovaSeq6000 (Illumina, San Diego, CA, USA).

The sRNA fraction was sequenced using one replicate per each time point; in order to preserve the integrity of the transcripts, no rRNA depletion was performed and the fragmentation step was skipped.

After quality control evaluation with FastQC [82], the reads were mapped on the reference *M. smegmatis* genome (NC_008596.1, http://www.ncbi.nlm.nih.gov/, accessed on 18 October 2022) using Bowtie2 [83]; the alignment was performed with the “-local” and “-dovetail” options. Calculation of the mapped fragments for all genes was performed with the featureCounts program from the Subread package [84]. Only unambiguously mapped non-chimeric fragments were used in subsequent analysis.

Differentially expressed genes (DEGs) were identified using the DESeq2 software package [85]. The genes were considered differentially expressed if the adjusted p value was ≤ 0.01 and the log2 fold change (log2FC) value was ≥ 1.

Gene Ontology (GO) analysis was performed using the Database for Annotation Visualization and Integrated Discovery (DAVID) online service to identify overrepresented functional categories among the identified clusters [86].

### 4.5. sRNA Annotation

The detection of putative sRNAs was performed with the Rockhopper software [87]. Potential sRNA genes were annotated in each sample (H0, H2, H5, and H24) and then merged into a consolidated annotation with duplicates collapsed; the genes annotated as encoding transposases were removed. To compare sRNA expressions between samples, the whole transcriptome data were re-analyzed using the pipeline on the consolidated sRNA annotation mentioned above. The annotation system for sRNAs was implemented following the template and recommendations provided in the paper by Lamichhane et al [15].

### 4.6. Quantitative Real-Time PCR (qRT-PCR)

cDNA was synthesized in reactions containing 1 µg of total RNA, random hexanucleotides (Evrogen, Moscow, Russia), and SuperScript III reverse transcriptase (Invitrogen, Carlsbad, CA, USA). qRT-PCR was performed with qPCRmix-HS SYBR (Evrogen, Moscow, Russia) in the LightCycler 480 real-time PCR system (Roche, Basel, Switzerland) at the following conditions: 40 cycles at 95 °C for 20 s, 60 °C for 20 s, and 72 °C for 30 s; primers are listed in Suppl. Table S6. At the end of amplification, a dissociation curve was plotted to confirm the specificity of the product. All qRT-PCR analyses were performed in triplicate, and the results were normalized against 16S rRNA to correct for sample-to-sample variations.

### 4.7. Data Presentation and Statistical Analysis

Time-dependent clustering of gene expression patterns was performed with the TCseq R package [88] only for those genes which were identified as differentially expressed in at least one of the five performed comparisons, and a heatmap was constructed using the bioinformatics.com.cn platform. Volcano plots were generated using the EnhancedVolcano R package [89].

Statistical analysis was performed with GraphPad Prism 8.0 (GraphPad Software Inc., La Jolla, CA, USA). The data were expressed as the mean ± SD. For non-normally distributed data, the Mann–Whitney U test was used. Differences were considered statistically significant at *p* < 0.05. At least three independent experiments were performed for each assay.

The secondary structures of sRNA were predicted using the RNAfold web server [90], and the resulting structures were visualized and drawn using the Visualization Applet for RNA (VARNA) package [91].

## 5. Conclusions

**Figure 7:**
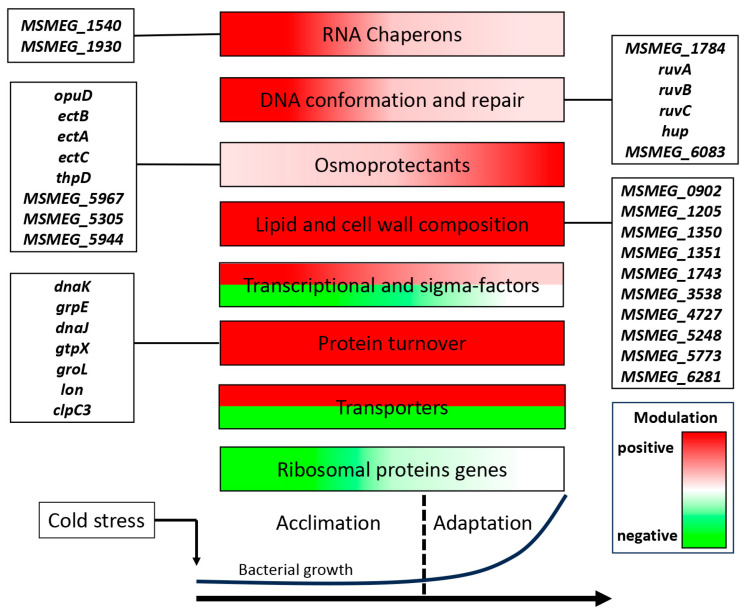
Schematic representation illustrating the modulation of functional gene groups in *M. smegmatis* under cold stress conditions.

In this study, we performed comprehensive transcriptomics of *M. smegmatis* exposed to cold stress and revealed two phases of expression changes corresponding to acclimation and adaptation stages. Time-dependent DEG clustering indicated that at the acclimation stage, the genes associated with responses to various stresses (cell wall remodeling, starvation, osmotic pressure, and oxidation) were upregulated, whereas the genes responsible for rapid growth were downregulated, thus providing resources to support bacterial survival in adverse conditions. In contrast, at the adaptation stage, most of the genes activated at the first stage were repressed, whereas many of the inhibited ones were re-activated. These changes in RNA level are schematized in Figure 7. The two-stage response of *M. smegmatis* to cold stress discovered in this study indicates a sophisticated regulatory system supporting its survival at low temperatures. Comparison of the adaptation mechanisms existing in *M. smegmatis* with those observed in model bacteria such as *E. coli* and *B. subtilis* revealed significant differences, emphasizing specialized strategies developed by *M. smegmatis* and possibly other mycobacteria, including MTB, to successfully endure cold stress. Further insights into the adaptive molecular mechanisms of mycobacteria should be gained by analyzing cold-induced proteins and their functional activities in the maintenance of cellular functions under stress conditions.

## Figures and Tables

**Table 1:** Selected DEGs upregulated by cold stress in *M. smegmatis*.

Gene	Process	Expression Pattern (Fold Change vs. H0)			Gene Product
		H2	H5	H24	
*MSMEG_1540*	RNA chaperones	9.71	2.04	3.49	ATP-dependent RNA helicase
*MSMEG_1930*		5.01	2.52	N/A	DEAD/DEAH box helicase
*MSMEG_6159* (*CspA*)		2.14	3.41	2.31	Putative cold shock protein A
*MSMEG_1784*	DNA conformation and repair	4.68	N/A	N/A	Type I topoisomerase
*MSMEG_2389* (*hup*)		1.54	2.39	N/A	DNA-binding protein HU
*MSMEG_2943* (*ruvC*)		2.47	1.79	N/A	Crossover junction endodeoxyribonuclease
*MSMEG_2944* (*ruvA*)		3.39	2.20	N/A	Holliday junction DNA helicase
*MSMEG_2945* (*ruvB*)		2.47	1.91	N/A	Holliday junction DNA helicase
*MSMEG_6083*		N/A	3.10	4.41	Base excision DNA repair protein
*MSMEG_1883* (*opuD*)	Osmoprotection	2.85	2.79	N/A	Glycine betaine transporter
*MSMEG_3898* (*thpD*)		5.45	3.62	7.29	Ectoine hydroxylase (ectoine biosynthesis)
*MSMEG_3899* (*ectC*)		4.11	2.46	7.68	Ectoine synthase (ectoine biosynthesis)
*MSMEG_3900* (*ectB*)		4.03	2.39	6.05	Diaminobutyrate--2-oxoglutarate aminotransferase (ectoine biosynthesis)
*MSMEG_3901* (*ectA*)		2.72	N/A	2.88	L-2,4-diaminobutyric acid acetyltransferase (ectoine biosynthesis)
*MSMEG_5967*		5.36	2.82	2.26	Glucose-methanol-choline oxidoreductase (choline metabolism)
*MSMEG_5305*		4.66	3.98	14.09	Choline dehydrogenase (choline metabolism)
*MSMEG_5944*		3.36	N/A	N/A	Glucose-methanol-choline oxidoreductase (choline metabolism)
*MSMEG_1878*	Translation	3.09	3.05	5.97	Mycobacteria-specific protein Y
*MSMEG_0709* (*dnaK*)	Protein turnover	2.96	2.26	2.67	Chaperone
*MSMEG_0710* (*grpE*)		2.47	2.08	2.22	Co-chaperone
*MSMEG_0711* (*dnaJ*)		2.70	2.77	2.46	Chaperone
*MSMEG_1134* (*htpX*)		3.99	1.81	N/A	Putative protease
*MSMEG_1978* (*groL*)		4.57	2.29	13.16	Chaperonin
*MSMEG_3582* (*lon*)		5.21	2.60	N/A	ATP-dependent protease La
*MSMEG_3761* (*clpC3*)		10.94	4.57	4.31	Clp protease subunit
*MSMEG_0902*	Cell wall and lipid composition	N/A	2.67	2.70	Cyclopropane-fatty-acyl-phospholipid synthase 1 (modification of mycolic acids)
*MSMEG_1205*		16.02	10.54	4.50	
*MSMEG_1350*		N/A	1.83	2.75	
*MSMEG_1351*		2.75	4.53	9.26	
*MSMEG_1743*		5.45	N/A	N/A	Fatty acid desaturase (desaturation of fatty acids)
*MSMEG_3538*		3.61	2.08	2.54	Cyclopropane-fatty-acyl-phospholipid synthase 1 (modification of mycolic acids)
*MSMEG_4727*		N/A	2.01	4.86	Mycocerosic acid synthase (synthesis of lipooligosaccharides)
*MSMEG_5248*		2.12	N/A	1.68	Acyl-[ACP] desaturase (desaturation of fatty acids)
*MSMEG_5773*		3.90	3.01	3.37	Fatty acid desaturase (desaturation of fatty acids)
*MSMEG_6281*		N/A	2.15	4.60	N-acetylmuramoyl-L-alanine amidase (cell wall hydrolase)

Upregulated genes are highlighted red. N/A, no change.

## Data Availability

All RNA-seq data generated in this study are publicly available in the Gene Expression Omnibus (GEO) database under accession number GSE232901.

## Supplementary Materials

The following supporting information can be downloaded at: https://www.mdpi.com/article/10.3390/ijms241612706/s1.
