# Supplementary figures and images for: Dynamic Transcriptional Landscape of Mycobacterium smegmatis under Cold Stress
Source: Int J Mol Sci. 2023 Aug 11;24(16):12706. doi: 10.3390/ijms241612706 (PMC10454040; doi:10.3390/ijms241612706)

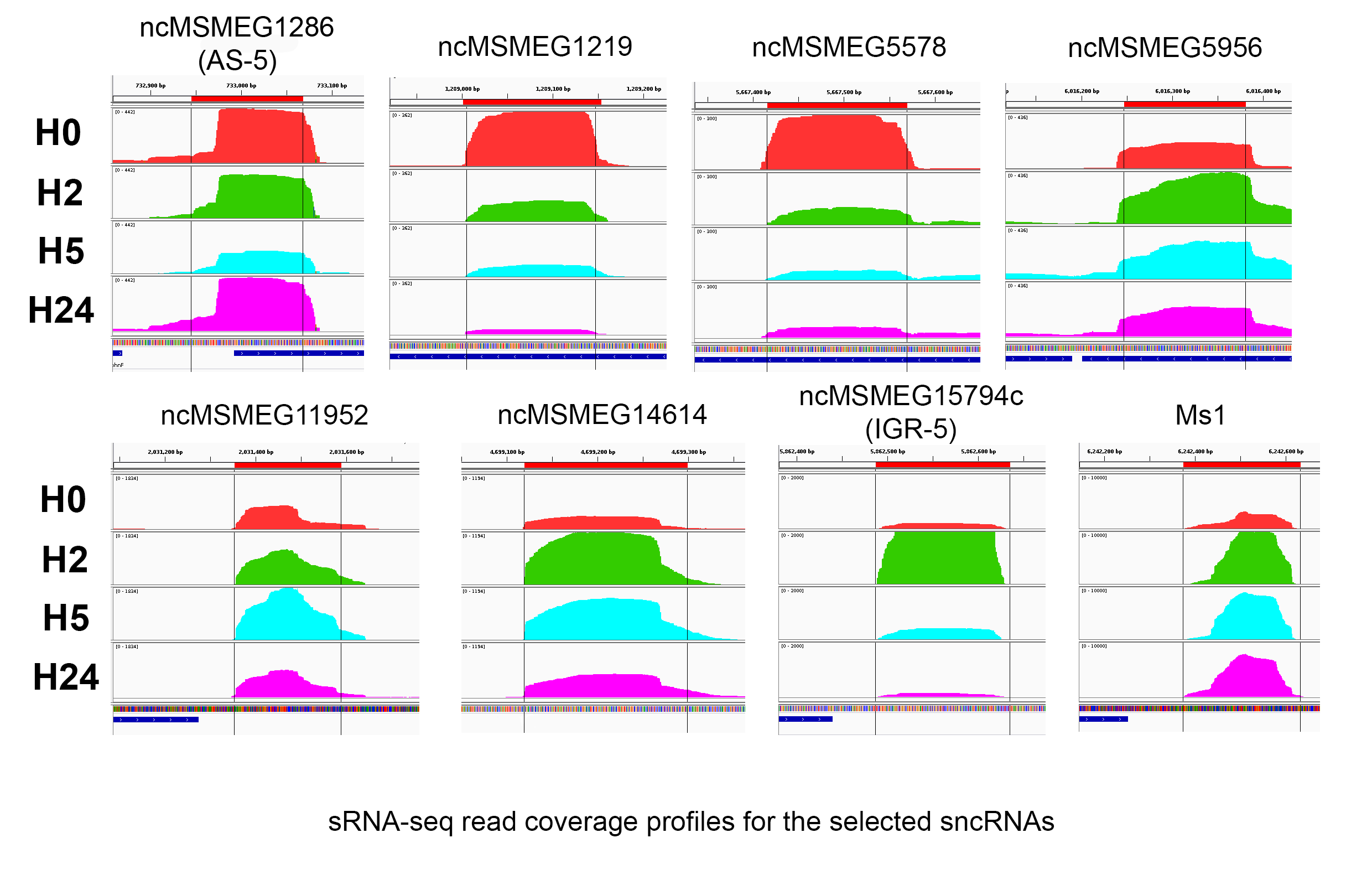

Supplement: Supplementary file 1 [file ijms-24-12706-s001.zip › Suppl_figure_S1.tif]

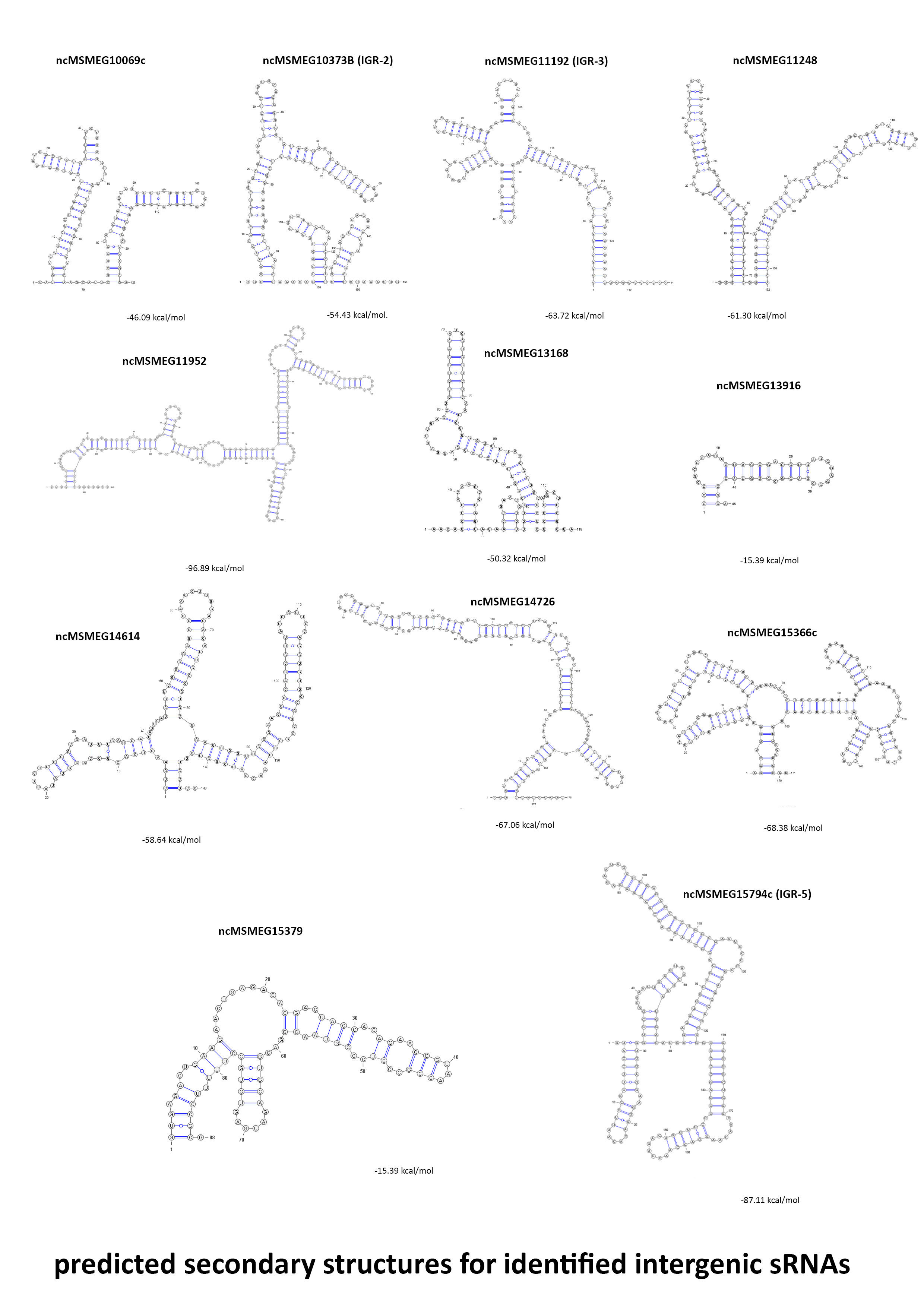

Supplement: Supplementary file 1 [file ijms-24-12706-s001.zip › Suppl_figure_S2.tif]
